# Therapeutic efficacy of budesonide suspension combined with poractant alfa injection for neonatal respiratory distress syndrome and its effect on serum ferritin and PAI-1 expressions

**DOI:** 10.12669/pjms.41.5.10496

**Published:** 2025-05

**Authors:** Yunbo Xu, Wenchao Chen

**Affiliations:** 1 Yunbo Xu Department of Neonatal Pediatrics, Huangshi Maternity and Children’s Health Hospital, Affiliated Maternity and Children’s Health Hospital of Hubei Polytechnic University, Huangshi Key Laboratory of Birth Defects Prevention, Huangshi 435000, China; 2 Wenchao Chen Department of Neonatal Pediatrics, Huangshi Maternity and Children’s Health Hospital, Affiliated Maternity and Children’s Health Hospital of Hubei Polytechnic University, Huangshi Key Laboratory of Birth Defects Prevention, Huangshi 435000, China

**Keywords:** Budesonide, Neonatal respiratory distress syndrome, PAI-1, Poractant alfa injection, Serum ferritin

## Abstract

**Objective:**

To investigate the therapeutic efficacy of budesonide suspension combined with poractant alfa injection for neonatal respiratory distress syndrome (NRDS) and the underlying action mechanisms.

**Methods:**

Sixty NRDS patients treated in Huangshi Maternity and Children’s Health Hospital from May 2021 to May 2023 were enrolled and were divided into control and observation groups, which were treated with poractant alfa injection and budesonide suspension combined with poractant alfa injection, respectively.

**Results:**

After 72 hours of treatment, the total efficacy in observation group was significantly higher than control group. Compared with control treatment, in observation group the partial pressure of oxygen was significantly increased, the partial pressure of carbon dioxide, peak inspiratory pressure, respiratory rate, fraction of inspired oxygen and serum tumor necrosis factor-α, interleukin-6, high-sensitivity C-reactive protein, serum ferritin and plasminogen activator inhibitor-1 levels were significantly decreased.

**Conclusion:**

Compared with poractant alfa injection, budesonide suspension combined with poractant alfa injection is more effective in treatment of NRDS. The action mechanisms may be related to its further reduction of inflammatory response and inhibition of serum ferritin and plasminogen activator inhibitor-1 expressions.

## INTRODUCTION

Neonatal respiratory distress syndrome (NRDS) often occurs in premature infants, and it is a common critical disease in the neonatal period. The lung hypoplasia and insufficient secretion of pulmonary surfactant (PS) cause the alveolar collapse and atrophy, affecting the lung ventilation and ventilation function, leading to NRDS. The manifestations of NRDS are dyspnea, respiratory failure, groan, cyanosis, etc. NRDS is one of the main causes of premature infant death.^1^,^2^ Clinically, exogenous PS is often used to treat NRDS.^3^ Poractant alfa injection is the common used exogenous PS. Its main components are phosphatidylcholine and specific protein, which can expand the alveoli and promote the synthesis and secretion of endogenous PS in the alveoli, thus promoting the development of the lung.^4^

Poractant alfa injection can alleviate the clinical symptoms of NRDS to a certain extent. However, this drug cannot be evenly distributed in the alveoli when administered through the trachea, which may increase the risk of lung injury.^5^ Budesonide is a common inhaled corticosteroid for local use. It has advantages of rapid action, small dosage and accurate action location.^6^ In addition, budesonide has little effect on systemic metabolism, with short half-life. It can relieve the pulmonary edema, reduce the inflammatory response, promote the vasoconstriction, reduce the exudate cells, and prevent the airway remodeling.^7^,^8^ Budesonide is also widely used in treating the respiratory tract disease. However, the combined use budesonide and poractant alfa injection for treating NRDS is not commonly reported. It is hypothesized that there is a synergistic effect between budesonide and poractant alfa injection. The purpose of this study was to investigate the therapeutic efficacy of budesonide suspension combined with poractant alfa injection for NRDS and the underlying action mechanisms.

## METHODS

This study collected the data of sixty NRDS patients treated in Huangshi Maternity and Children’s Health Hospital (Huangshi, China) from May 2021 to May 2023. The patients were divided into control group (30 cases) and observation group (30 cases) according to treatment methods. In the control group, there were 18 males and 12 females. The gestational age was 29.45±1.62 weeks. The body weight at birth was 1304.56±123.42 g. As for delivery mode, there were vaginal delivery in 22 cases and cesarean section in 8 cases. As for NRDS grading, there were Grade-III in 17 cases and Grade-IV in 13 cases. In the observation group, there were 16 males and 14 females. The gestational age was 30.07±1.84 weeks.

The body weight at birth was 1359.56±139.60 g. As for delivery mode, there were vaginal delivery in 19 cases and cesarean section in 11 cases. As for NRDS grading, there were Grade-III in 20 cases and Grade-IV in 10 cases. The general data had no statistically significant difference between two groups (P > 0.05).

### Ethical Approval:

This study was approved by the ethics committee of Huangshi Maternity and Children’s Health Hospital (No. 2024-LWSC-003; Date: June 4, 2024). Written informed consent was obtained from all participants.

### Inclusion criteria:

- The gestational age was less than 32 weeks
- The disease complied with diagnostic criteria of NRDS
- The disease occurred within 24 hours after birth
- The disease conformed to the treatment indication of mechanical ventilation.

### Exclusion criteria:

- The patients were complicated with intrauterine infectious pulmonary disease.
- The patients were complicated with congenital heart disease
- The patients were complicated with congenital respiratory malformation.

### Treatment methods:

Both groups of newborns received the basic interventions such as nutritional support, anemia correction, airway cleaning, infection prevention, sputum aspiration, and stabilization of internal environment, and received the mechanical ventilation. The control group was treated with poractant alfa injection (Chiesi Farmaceutici S.p.A. Chiesi group, Italy; 3 ml: 0.24 g) at dose of 100 mg/kg, once a day. The drug was preheated to 37ºC and fully shaken before administration, and the drug was extracted with a sterile syringe and dropped into the air tube. On the basis of the above treatment, the observation group was treated with inhalation of budesonide suspension (AstraZeneca Pty Ltd.; 2 ml: 0.5 mg), with dose of 0.25 mg/kg. The drug was dissolved in 0.9% sodium chloride solution for atomization inhalation, 5-10 min/time, once a day. The administration was stopped when the endotracheal tube was pulled out or the fraction of inspired oxygen (FiO_2_) was less than 40%. Both groups were treated for 72 hours. The adverse reactions were observed during treatment.

### Evaluation of clinical efficacy:

After 72 hours of treatment, the clinical efficacy in two groups was evaluated as follows: remarkably effect: the symptoms completely disappeared, the blood gas indicators returned to normal, and the X-ray showed clear lung texture; effective: the symptoms were relieved, the blood gas indicators were improved, and the X-ray showed lung abnormalities; ineffective: all indicators were not improved or were aggravated. The total effective rate was calculated as follows: total effective rate (%) = (number of markedly effective cases + number of effective cases) / total number of cases × 100%.

### Blood gas analysis:

Before and after treatment, the artery blood was collected from the patients, and put into anticoagulant tube containing heparin. The partial pressure of oxygen (*p*O_2_) and partial pressure of carbon dioxide (*p*CO_2_) were measured using the blood gas analyzer.

### Recording of ventilator parameters:

Before and after treatment, the ventilator parameters including peak inspiratory pressure (PIP), respiratory rate (RR) and FiO_2_ in two groups were recorded.

### Detection of blood biochemical parameters:

Before and after treatment, the fasting venous blood was taken from the patients. After centrifuging at 3000 rpm for 10 minutes, the serum was obtained. The inflammatory response indexes including tumor necrosis factor-α (TNF-α), interleukin-6 (IL-6) and high-sensitivity C-reactive protein (hs-CRP), serum ferritin (SF) and plasminogen activator inhibitor-1 (PAI-1) were detected by enzyme-linked immunosorbent assays. The detection operations followed the instructions of kits.

### Statistical analysis:

Data process was performed using SPSS 22.0 software. The measurement data were presented by mean±standard deviation, with comparison between two groups using t test. The enumeration data were presented by number or rate, with comparison between two groups using Chi-square test. P < 0.05 was considered as statistically significant.

## RESULTS

As shown in Table-I, after 72 hours of treatment, there were 16 markedly effective, six effective and eight ineffective cases in control group, with total effective rate of 73.33%. In observation group, there were 20 markedly effective, eight effective and two ineffective cases, with total effective rate of 93.33%. The total effective rate in observation group was significantly higher than that in control group (χ^2^ = 4.320; P = 0.038).

**Table-I:** Comparison of clinical efficacy between two groups.

Group	n	Markedly effective (n)	Effective (n)	Ineffective (n)	Total effective rate (%)
Control	30	16	6	8	73.33
Observation	30	20	8	2	93.33
*χ^2^*					4.320
*P*					0.038

Before treatment, there was no significant difference of *p*O_2_ or *p*CO_2_ between control and observation groups (P > 0.05). After treatment, compared with before treatment, in each group the *p*O_2_ was significantly increased (P < 0.05), and the *p*CO_2_ was significantly decreased (P < 0.05). Compared with control treatment, in observation group the *p*O_2_ was further increased (P < 0.05), and the *p*CO_2_ was further decreased (P < 0.05) (Table-II).

**Table-II:** Comparison of blood gas indexes between two groups.

Index	Group	n	Before treatment	After treatment	t	P
*p*O_2_ (mmHg)	Control	30	44.09±5.04	70.36±8.05	15.150	0.000
	Observation	30	46.12±4.62	79.52±6.47	23.011	0.000
	t		1.626	4.858		
	P		0.109	0.000		
*p*CO_2_ (mmHg)	Control	30	59.05±6.03	45.16±4.32	10.256	0.000
	Observation	30	56.73±5.59	41.25±3.06	13.305	0.000
	t		1.545	4.045		
	P		0.128	0.000		

*p*O_2_, partial pressure of oxygen; *p*CO_2_, partial pressure of carbon dioxide.

Table-III shows that, the ventilator parameters including PIP, RR and FiO_2_ had no obvious difference between two groups, respectively (P > 0.05). After treatment, compared with before treatment, in two groups each index was significantly decreased, respectively (P < 0.05). In addition, each index in observation group was significantly lower than that in control group (P < 0.05).

**Table-III:** Comparison of ventilator parameters between two groups.

Index	Group	n	Before treatment	After treatment	t	P
PIP (mmH_2_O)	Control	30	23.17±4.73	20.18±3.63	2.747	0.008
	Observation	30	22.56±3.18	18.05±2.40	6.200	0.000
	*t*		0.586	2.681		
	*P*		0.560	0.010		
RR (beats/min)	Control	30	48.81±7.05	42.84±6.57	3.393	0.001
	Observation	30	46.42±9.15	36.25±7.29	4.761	0.000
	*t*		1.133	3.678		
	*P*		0.262	0.001		
FiO_2_ (%)	Control	30	52.73±11.26	34.77±6.94	7.437	0.000
	Observation	30	51.81±9.04	28.40±5.22	12.283	0.000
	*t*		0.349	4.018		
	*P*		0.728	0.000		

PIP, peak inspiratory pressure; RR, respiratory rate; FiO_2_, fraction of inspired oxygen.

Before treatment, the serum TNF-α, IL-6 and hs-CRP levels had no significant difference between two groups (P > 0.05). After treatment, each index in two groups was significantly lower than that before treatment (P < 0.05), and that in observation group was significantly lower than that in control group (P < 0.05) (Table-IV).

**Table-IV:** Comparison of inflammatory response indexes between two groups.

Index	Group	n	Before treatment	After treatment	t	P
TNF-α (pg/ml)	Control	30	65.98±9.63	42.05±7.06	10.977	0.000
	Observation	30	68.73±12.27	31.17±6.16	14.984	0.000
	*t*		0.966	6.360		
	*P*		0.338	0.000		
IL-6 (pg/ml)	Control	30	30.13±7.20	22.79±5.06	4.568	0.000
	Observation	30	28.50±6.16	14.24±2.87	11.493	0.000
	*t*		0.942	8.050		
	*P*		0.350	0.000		
hs-CRP (μg/ml)	Control	30	12.21±3.08	7.05±1.78	7.945	0.000
	Observation	30	13.37±2.8	5.82±1.23	13.244	0.000
	*t*		1.509	3.114		
	*P*		0.137	0.003		

TNF-α, tumor necrosis factor-α; IL-6, interleukin-6; hs-CRP, high-sensitivity C-reactive protein.

The SF and PAI-1 levels had no obvious difference between two groups, respectively (P > 0.05) is shown in Table-V. After treatment, compared with before treatment, in two groups each index was significantly decreased, respectively (P < 0.05). In addition, each index in observation group was significantly lower than that in control group (P < 0.05). The mechanical ventilation time, ventilator withdrawal time and hospital stay in observation group were significantly shorter than those in control group, respectively (P < 0.05) is presented in Table-VI.

**Table-V:** Comparison of SF and PAI-1 levels between two groups.

Index	Group	n	Before treatment	After treatment	t	P
SF (ng/ml)	Control	30	167.34±32.74	123.09±23.10	6.053	0.000
	Observation	30	180.21±40.27	108.30±20.21	8.746	0.000
	*t*		1.358	2.645		
	*P*		0.180	0.010		
PAI-1 (ng/ml)	Control	30	48.15±12.42	24.87±5.05	9.510	0.000
	Observation	30	52.58±11.62	21.28±4.20	13.875	0.000
	*t*		1.427	2.994		
	*P*		0.159	0.004		

SF, serum ferritin; PAI-1, plasminogen activator inhibitor-1.

**Table-VI:** Comparison of mechanical ventilation time, ventilator withdrawal time and hospital stay between two groups.

Group	n	Mechanical ventilation time (days)	Ventilator withdrawal time (days)	Hospital stays (days)
Control	30	4.78±0.87	26.98±5.53	47.48±8.16
Observation	30	4.19±0.77	22.16±4.17	41.59±8.05
*t*		2.781	3.812	2.814
*P*		0.007	0.000	0.007

During the treatment, there were three cases of ventilator-associated pneumonia, three cases of pulmonary hemorrhage, two cases of septicemia and two cases of necrotizing enterocolitis in control group, with two cases of ventilator-associated pneumonia, two cases of pulmonary hemorrhage, one case of septicemia and two cases of necrotizing enterocolitis in observation group. There was no significant difference of adverse reaction incidence between two groups (33.33% vs. 23.33%; χ^2^ = 0.739; P = 0.390) (Table-VII).

**Table-VII:** Comparison of adverse reactions between the two groups.

Group	n	Ventilator-associated pneumonia	Pulmonary hemorrhage	Septicemia	Necrotizing enterocolitis	Incidence (%)
Control	30	3	3	2	2	33.33
Observation	30	2	2	1	2	23.33
*χ^2^*						0.739
*P*						0.390

## DISCUSSION

This study investigated the therapeutic efficacy of budesonide suspension combined with poractant alfa injection for NRDS and the underlying action mechanisms. Results showed that, after 72 hours of treatment, the total efficacy in observation group was significantly higher than control group. Compared with control treatment, in observation group the partial pressure of oxygen was significantly increased, the partial pressure of carbon dioxide, peak inspiratory pressure, respiratory rate, fraction of inspired oxygen and serum tumor necrosis factor-α, interleukin-6, high-sensitivity C-reactive protein, serum ferritin and plasminogen activator inhibitor-1 levels were significantly decreased.

NRDS is the main cause of premature infant death. PS preparation is the first choice for clinical prevention and treatment of NRDS.^9^ In addition, the continuous oxygen therapy, artificial airway mechanical ventilation and other measures are taken for NRDS, but these measures may increase the risk of bronchopulmonary dysplasia. Budesonide is a new glucocorticoid preparation, and has the advantages of small dosage, rapid action and accurate action site, and is gradually used in clinical practice.^10^ Previous study has confirmed the efficacy of budesonide combined with poractant alfa for respiratory distress syndrome in animal model.^11^ In this study, after treatment, the total effective rate in observation group was significantly higher than that in control group. Compared with control treatment, in observation group the *p*O_2_ was significantly increased, and the *p*CO_2_ was significantly decreased. It is suggested that, compared with single use of poractant alfa injection, budesonide suspension combined with poractant alfa injection can improve the therapeutic efficacy in treatment of NRDS. This is consistent with the above research results.

In addition, the ventilator parameters including PIP, RR and FiO_2_ in observation group were significantly lower than control group. This also reflects the advantage of this combined medication strategy. There was no significant difference of adverse reaction incidence between two groups. This indicates that budesonide suspension combined with poractant alfa injection has good safety. Progression of NRDS is closely related to the inflammatory response.^12^ TNF-α is secreted by activated mononuclear macrophages. It can mediate the body’s inflammatory response.^13^ IL-6 is mainly produced by T lymphocytes and B lymphocytes. It plays a regulatory role in inflammatory response.^14^ hs-CRP is an inflammatory factor index, and its content increases significantly when the tissue is injured.^15^ Both budesonide and poractant alfa can reduce the inflammatory response in body.^16^,^17^ Results of this study showed that, after treatment, the serum TNF-α, IL-6 and hs-CRP levels in observation group were significantly lower than control group. This suggests that, compared with single use of poractant alfa injection, the combined use of budesonide suspension and poractant alfa injection can further reduce the inflammatory response, which may be related its improvement of therapeutic efficacy for NRDS.

SF is synthesized by reticuloendothelial cells and hepatocytes, and has the function of tissue repair and immunosuppression.^18^ The serum SF level can reflect the degree of inflammation and lung injury, and is closely related to the occurrence and development of respiratory distress syndrome.^19^ PAI-1 and urokinase-type plasminogen activator (uPA) are important members of the fibrinolytic family. If they are out of balance, the exudates in the alveoli will not be cleared, and the fibroblasts and other cells produce new matrix proteins to form scars, leading to the lung injury.^20^ It is found that, high concentrations of PAI-1 and an increased ratio of PAI-1 to uPA, with a concurrently less-increased ratio of PAI-1 to tPA, are associated with the severity of NRDS during the first postnatal days.^21^ It is found that, budesonide can lower the PAI-1 lever in rats with airway inflammation.^22^ In our study, after treatment, compared with before treatment, in each group the SF and PAI-1 levels were significantly decreased. Compared with control group, in observation group each index was further decreased. This shows that budesonide suspension combined with poractant alfa injection can significantly reduce the expression level of SF and PAI-1, thus reducing lung injury and improving the lung function of patients with NRDS.

Findings of our study can provide an important basis for further application of budesonide suspension combined with poractant alfa injection to treating NRDS. There may be other action mechanisms of budesonide suspension and poractant alfa injection on NRDS, and they should be further explored.

### Limitations:

This study still has certain shortcomings, including relatively insufficient number of cases and insufficient follow-up time. Therefore, in next study, the larger sample size and longer follow-up visit are needed to verify the findings.

## CONCLUSIONS

Compared with single use of poractant alfa injection, budesonide suspension combined with poractant alfa injection is more effective in treatment of NRDS. The action mechanisms may be related to its further reduction of inflammatory response and inhibition of SF and PAI-1 expressions. This strategy has good safe, and is worthy of clinical application.

### Authors’ contributions:

**WC:** Designed the study. prepared the manuscript, is responsible and accountable for the accuracy or integrity of the work.

**YX:** Literature search, Collected and analyzed clinical data, and significantly revised this manuscript.
